# Pharmacological upregulation of GLT-1 alleviates the cognitive impairments in the animal model of temporal lobe epilepsy

**DOI:** 10.1371/journal.pone.0246068

**Published:** 2021-01-28

**Authors:** Daniel Ramandi, Mahmoud Elahdadi Salmani, Ali Moghimi, Taghi Lashkarbolouki, Masoud Fereidoni

**Affiliations:** 1 Faculty of Science, Department of Biology, Ferdowsi University of Mashhad, Mashhad, Iran; 2 School of Biology, Damghan University, Damghan, Iran; University of Modena and Reggio Emilia, ITALY

## Abstract

It is known that hippocampal epileptogenesis is accompanied by hyperexcitability, glutamate-related neuronal dysfunctions and consequently cognitive deficits. However, the neuroprotective role of astrocytic glutamate uptake through the Glutamate Transporter-1 (GLT-1) remains to be unknown in these processes. Therefore, to assess the effect of glutamate uptake, pharmacological upregulation of GLT-1 using ceftriaxone administration (200 mg/kg/day, i.p, 5 days) was utilized in Li-PIL animal models of temporal lobe epilepsy (TLE). Glutamate concentration and glutamine synthetase activity were analyzed using biochemical assays. In addition, GLT-1 gene expression was assessed by RT-qPCR. Finally, cognitive function was studied using Morris water maze (MWM) test and novel object recognition task (NORT). Our results demonstrated that the acute phase of epileptogenesis (first 72 hours after Status Epilepticus) was accompanied by an increase in the hippocampal glutamate and downregulation of GLT-1 mRNA expression compared to controls. Ceftriaxone administration in epileptic animals led to a reduction of glutamate along with elevation of the level of glutamine synthetase activity and GLT-1 expression in the acute phase. In the chronic phase of epileptogenesis (4 weeks after Status Epilepticus), glutamate levels and GLT-1 expression were decreased compared to controls. Ceftriaxone treatment increased the levels of GLT-1 expression. Furthermore, impaired learning and memory ability in the chronic phase of epileptogenesis was rescued by Ceftriaxone administration. This study shows that astrocytic glutamate uptake can profoundly impact the processes of hippocampal epileptogenesis through the reduction of glutamate-induced excitotoxicity and consequently rescuing of cognitive deficits caused by epilepsy.

## Introduction

Epilepsy is regarded as the occurrence of two or more spontaneous recurrent seizures [1]. It is well established that epileptic individuals exert cognitive dysfunction [2]. Investigation of the animal models of temporal lobe epilepsy can suitably demonstrate these cognitive deficits and play an important role in elucidating the mechanism underlying hippocampal epileptogenesis, thus aiding in the development of new drugs and therapies. Temporal lobe epileptogenesis has been extensively studied in two main phases. The early phase of epileptogenesis, known as the acute phase is accompanied by neuroinflammation, excitotoxic apoptosis and changes in synaptic activity. This initial phase is followed by 2–4 weeks of latent period and beginning of the chronic phase of epileptogenesis. The chronic phase is characterized by hyperexcitability of neuronal networks, hippocampal mossy fiber sprouting and finally, hippocampal sclerosis [3]. Glutamate excitotoxicity has been proposed as the main consequence of seizure and early epileptogenesis [4]. Successive neuronal firing, as well as malfunctioning of glutamate clearance by astrocytes, can increase the extracellular concentration of this excitatory neurotransmitter and cause hyperexcitability in the neural circuits which can, in turn, affect many behavioral functions including but not limited to learning and memory [5]. Long-term potentiation (LTP) and depression (LTD) have been extensively studied as the main cellular correlates of learning and memory [6]. Interruptions and malfunctioning of these phenomena in the hippocampus are seen in most of the neurological disorders consisting of cognitive disabilities [7–9]. In epileptic individuals who suffer from temporal lobe epilepsy, as well as in animal models, learning and memory impairments have been reported which is probably due to excitotoxicity, hyperexcitability, impairment of LTP/LTD balance and cell death [2].

Astrocytes due to their abundance of membrane channels, receptors and transporters have implicated in many neurophysiological processes including synaptic reorganization, extracellular ion and metabolite hemostasis, vascular tone regulation and neuronal synchronization [10]. Astrocytic glutamate uptake through glutamate transporter 1 (GLT-1) has been shown to play an important role in seizure sensitivity [11]. The clearance of glutamate is crucial not only in neuronal excitability but also in the accurate regulation of the glutamate-glutamine cycle [12]. The glutamate cleared by astrocytes is converted to glutamine by the enzyme glutamine synthetase and is delivered to the adjacent neurons for glutamate and/or GABA production [13].

Ceftriaxone is a third-generation cephalosporin that has proved to be useful in many disorders of the nervous system by inhibiting excitotoxicity through astrocytic GLT-1 upregulation [14]. It can induce transcription of the GLT-1 gene by activating the NF-κB (nuclear factor kappa-light-chain-enhancer of activated B cells) [15]. Many research has confirmed its beneficial effects on traumatic brain injury [16], cerebral ischemia [17] and epilepsy [18]. The fact that one of the worsening effects of epilepsy is due to a decrease in GLT-1 expression and thus increasing the extracellular glutamate levels, give rise to the hypothesis that astrocytic glutamate uptake has an important role in the progression of epileptogenesis and it could be a valuable target in anti-epileptic therapies.

Therefore, it seems that pharmacological upregulation of GLT-1 and decreasing excitotoxicity might reverse some, if not all, of the molecular and behavioral consequences of seizures and epilepsy. In this study, we investigated the beneficial effects of increased astrocytic glutamate uptake through GLT-1 on hippocampal epileptogenesis and cognitive dysfunctions induced by epilepsy. It is demonstrated that excitotoxicity in the early acute phase of epileptogenesis is one of the key inceptions of downstream neuroinflammatory and apoptotic processes and subsequent cognitive deficits in epileptic individuals.

## Materials and methods

Here, we investigated the effects of GLT-1 upregulation on cognitive functions and excitotoxicity in the animal models of temporal lobe epilepsy (TLE), using behavioral, molecular and biochemical assays.

### Animals

Male Wistar rats (200–280 g) were used for this experiment. The animals were housed under standard conditions including constant temperature (22–24°C) and humidity (45–60%), 12 h light/dark cycle, and access to food and water *ad libitum*. Procedures involving animals and their care were carried out in accordance with National Institute of Health Guide for the Care and Use of Laboratory Animals (NIH Publication No. 23–80, revised 1996), national and local approved guidelines and policies. All experimental protocols were approved by the Ferdowsi University of Mashhad Ethical Committee for Animal Experimentation and by the Iranian Ministry of Health. All animals were habituated to laboratory conditions and were used only once during the seizure induction protocol. The least number of animals was used following the ARRIVE (Animal Research: Reporting *In Vivo* Experiments) [19]. The number of animals used for each experiment is summarized in the respective table (Table 1).

**Table 1:** Number of animals used for each experimental procedure per group.

Experiment	Number of Animals per Group			
	Control	Pil	Pil+Cef	Cef
Seizure Scoring and Surveillance	-	25	25	-
Morris Water Maze	7	7	7	7
Novel Object Recognition Task	7	7	7	7
RT-qPCR	10	10	10	10
GS Activity	10	10	10	10
Glutamate Assay	10	10	10	10

### Groups and study design

**Fig 1:**
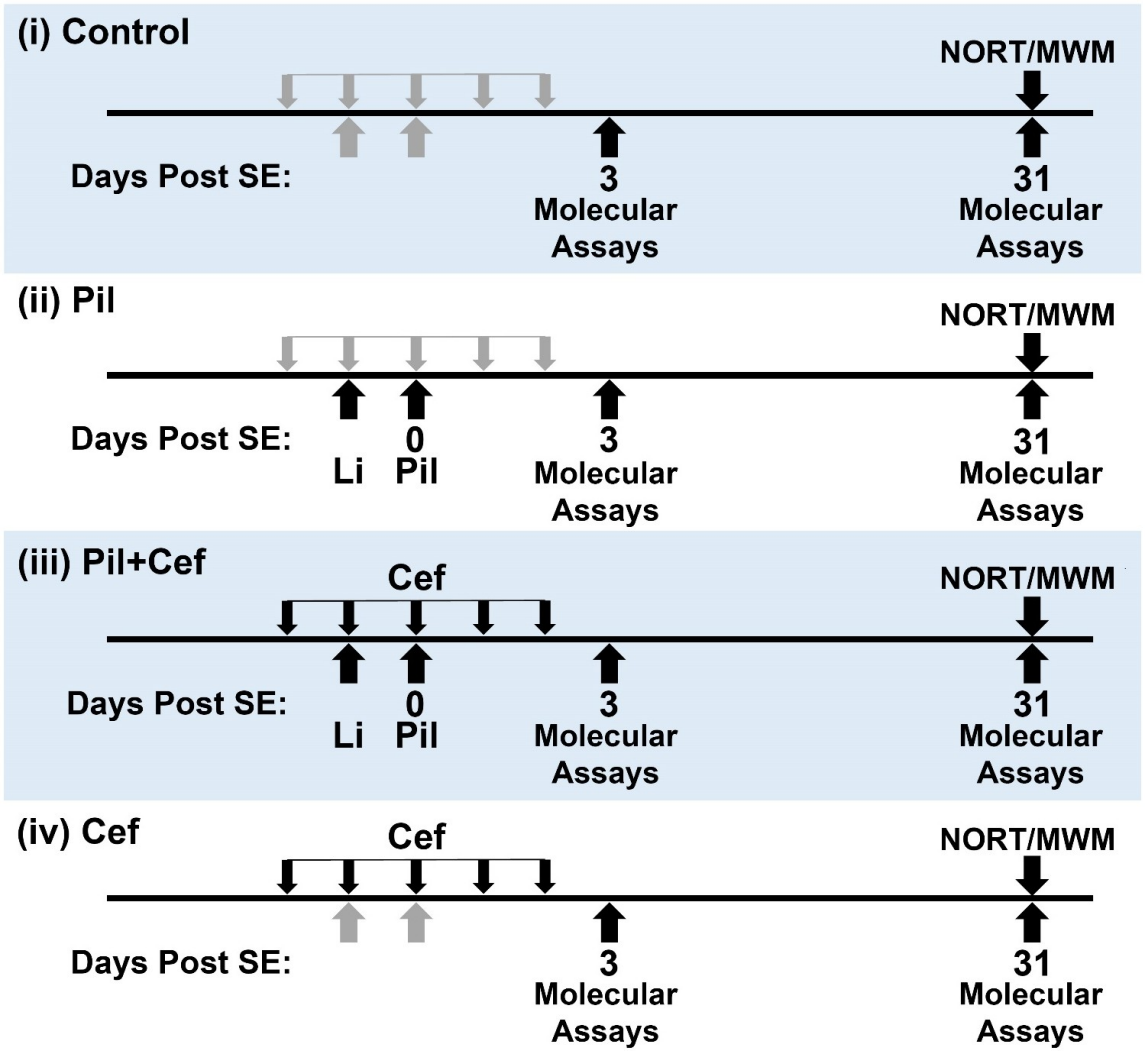
Study design and animal groups. The animals were randomly assigned to one of the four groups. SE, Status Epilepticus; NORT, Novel Object Recognition Task; MWM, Morris Water Maze; Li, Lithium administration; Pil, Pilocarpine; Cef, Ceftriaxone.

The animals were randomly assigned in the four studied groups (as shown in Fig 1): (i) Control animals received drug vehicles, (ii) Pil animals that received a single injection of Lithium Chloride (3 mEq/kg i.p.) 20 hours before pilocarpine, followed by methyl-scopolamine (1 mg/kg, s.c.) 45 minutes prior to pilocarpine (30 mg/kg, i.p.), (iii) Pil+Cef animals received Lithium, pilocarpine and methyl-scopolamine (same doses as Pil animals) along with 5 daily administrations of Ceftriaxone (200 mg/kg/day) starting at 48 hours before pilocarpine administration, and (iv) Cef animals were only subjected to 5 daily injections of Ceftriaxone. The animal’s behavior was observed by the researchers for 6 hours thereafter. Animals that developed seizures evolving into status epilepticus (SE) within the first 30 minutes after pilocarpine injection were included in the study. As previously described by Corda et. al [20], SE is defined as spontaneous recurrent generalized seizures. SE was interrupted 90 minutes after onset by an injection of diazepam (10 mg/kg, i.p.) followed by two injections of diazepam (2.5 mg/kg) 6 and 8 hours after SE induction respectively. To reduce the effect of diazepam and methyl-scopolamine on the study, these drugs were administered to all animals in all of the groups. Animals usually showed short-lasting seizures and weight loss during the first 3 days of epileptogenesis and were given special accessibility to food and water. This stage was followed by recovery and entering a period of apparent well-being.

### Seizure scoring and surveillance

Seizure scoring and surveillance was carried out using video monitoring as previously proposed [21] during the first day of SE induction and a seven-day period three weeks post SE. As previously shown [21], the latency to the first spontaneous motor seizure is miscalculated at most by 72 hours by video-monitoring as compared to electroencephalographic recordings (EEG). Thus, in the present study video-monitoring (8 hours per day) was preferred over EEG to avoid infections and complications caused by long-term behavioral tasks. Costa et. al (2020) have shown that the latency to develop SE is correlated with the latency to the first spontaneous recurrent seizure (SRS) [22]. To minimize any potential effects of the latency period, only the animals that showed SRS within 4 weeks after SE were selected for further behavioral and molecular studies. Seizures were scored by a trained experimenter blind to the study, using the stages previously described by Corda et. al [20] as follows: 0: No response, 1: facial jerks, 2: myoclonic seizures, 3: forelimb clonic seizures, 4: Generalized clonic seizures with rearing and falling, 5: recurrent generalized seizures (SE). SE latency, number and duration of 3rd and 4th stage seizures were studied in the animals. Animals used for the behavioral and molecular experiments were randomly chosen from the animals that developed SE.

#### Behavioral assays

***Morris Water Maze***. Animals (n = 7 in each group) were tested for spatial learning and memory in the Morris water maze (MWM) test 31 days after SE induction. The MWM circular basin was 150 cm in diameter and 70 cm in height, filled with water (24±1°C). Visual cues included both extramazal cues placed on the walls of the room and intramazal on the walls of the basin. The escape platform was 10 cm in diameter and was submerged 3 cm under water. The experiment was conducted as previously described with minor modifications [23]. The experiment was run in four phases: acquisition learning, probe trial for reference memory, reversal learning, probe trial for reversal learning and the visible platform task. On days 1 to 5 the hidden platform task was conducted to test acquisition learning. Four trials with 5-minute inter-trial intervals were conducted on each of the acquisition learning days. Animals were given 60 seconds to find the hidden platform. Rats finding the escape platform were left on it for 15 seconds while unsuccessful animals were assigned a 60 second latency and were guided to the platform and were left there for 15 seconds. A single one-minute probe trial was performed on day 6 during which the escape platform was removed from the pool and animals were released from the opposite quadrant where the platform was located. On days 7, 8 and 9, the reversal learning task was performed. For the reversal learning, the platform was relocated to the opposite quadrant relative to its position during acquisition learning. The reversal learning task was performed similar to acquisition learning. On day 10 a single reversal probe trial was conducted similar to the acquisition learning probe trial. To assess the ability of the animals to learn the task and check their visual ability, the visible platform task was conducted on days 11, 12 and 13 for four one-minute trials during which the platform location was changed for each trial. The platform was made visible using an aluminum foil and was elevated 3 cm above the water surface. Indirect dim lighting was used in the room where the maze was kept. The data were recorded using an analogue camera and analyzed using ANY-Maze 5.1 tracking software.

***Novel Object Recognition Task***. To assess object recognition memory, Novel Object Recognition Task (NORT) was used as previously described [24], 31 days post SE (n = 7 in each group). For this study, a three-compartment apparatus was used (dimension of the two end compartments: 31 x 24 x 45.5 cm; dimensions of the center compartment: 15 x 24 x 45.5 cm). Two inside walls of the center compartment, when raised, allowed the rats to move freely between the end compartments. The walls were lowered during the object exposure phase and was elevated in the habituation phase and the novel object test trial.

The test was conducted in three phases. In the first day, the habituation phase was performed, in which the inside walls were raised and the animal was left in the apparatus to move freely for 10 minutes. Isopropyl alcohol was used to clean the apparatus between every trial of each animal. 24 hours later, the object exposure phase (training) was performed. During this phase the inside walls were lowered and the familiar object was placed in either end compartments. The animal was given 5 minutes to explore the object. The object was then deodorized using Isopropyl alcohol and was put in the other compartment. Another 5 minutes was given to the animal to explore the object. 90 minutes after the training, the novel object recognition test was done during which a novel object different in both shape and color was put in one end compartment and the familiar object in the other. The animal was placed in the center with the inside walls raised and was left to freely explore objects for 10 minutes. The animal behavior during the testing phase was recorded using an analogue camera and analyzed by ANY-Maze 5.1 tracking software.

#### Molecular assays

***Reverse Transcriptase qPCR (RT-qPCR)***. To measure GLT-1 and GAPDH gene expression, rats were deeply anesthetized with 100% CO_2_, killed, and then hippocampus were quickly removed. RNA was extracted by conventional TRIzol method (RNX-Plus, SinaClone, Iran) from the hippocampus tissue either 72 hours or 31 days post SE. The concentration and integrity of extracted RNA was determined by UV spectrophotometry and gel electrophoresis. cDNA was synthesized using RevertAid First Strand cDNA Synthesis Kit (Thermo Fisher, USA). The primers were designed and synthesized by Macrogen, Inc. (Seoul, South Korea) and are available upon request. Reaction system was 2X SYBR Green PCR Master mix (Parstous, Iran) 12.5 μl + upstream and downstream primers (10 pmol/ul) 1 μl each + cDNA template 1 μl, adding water to the total volume of 25 ul. The reaction condition was the same for the both genes analyzed: an initial denaturation at 95°C for 2 min, and 40 cycles of 95°C for 15 sec, 58°C for 20 sec, 72°C for 25 sec. Amplification curves were constructed, and the relative expression of mRNA (GAPDH and b-Actin as the references) was calculated by 2-ΔΔCq method as previously described [25]. Primers used for the cDNA amplifications can be found in the respective table (Table 2).

**Table 2:** Primers used for the cDNA amplification (qPCR).

Gene	F/R	Tm	Primer Length	Sequence	Product Length
GLT-1	F	60.33	20	TGGACTGGCTGCTGGATAGA	93
	R	59.97	20	GCTCGGACTTGGAAAGGTGA	
GAPDH	F	60.68	20	AGTGCCAGCCTCGTCTCATA	133
	R	60.39	20	ATGAAGGGGTCGTTGATGGC	
β-Actin	F	60.03	20	CCACCATGTACCCAGGCATT	104
	R	59.6	20	CTATGGGTCCAGGCTAAGGC	

#### Biochemical assays

***Glutamine Synthetase activity***. The activity of glutamine synthetase was determined by measuring the rate of glutamine formation as previously described [26]. Hippocampal samples were homogenized using 1 ml ice cold PBS and the protein content was assessed using the Bradford protein assay. 0.1 ml of homogenized sample was added to 0.9 ml of a solution (at 37°) containing imidazole-HCl buffer (50 μmol), sodium ATP (10 μmol), MgCl_2_ (20 μmol), sodium L-glutamate (50 μmol), hydroxylamine (100 μmol), and 2-mercaptoethanol (25 μmol). After incubation at 37° for 15 minutes, ferric chloride solution (1.5 ml) was added. The precipitated protein was removed by centrifugation, and the absorbency at 535 nm was read against a reagent blank. Standard curve analysis showed that the rate of the enzymatic reaction was linear with enzyme concentration in the range of 0.2–1.2 μmol of hydroxamate formed. A unit of glutamine synthetase activity was defined as amount of enzyme which catalyzes the synthesis of 1 μmol of y-glutamyl hydroxamate under the conditions given above. Specific enzyme activity was expressed as units per milligram of total protein.

***Glutamate assay***. Glutamate content was examined as previously described [27]. Briefly, Glutamate dehydrogenase (0.225 U/50 μl) was added to Glycine-Hydrazine buffer (0.6 ml) containing the samples (50 μl), ADP (33.5 mM/30 μl) and NAD (27 mM/60 μl). NADH production in the reaction mixture was observed using UV spectroscopy as a decrease in 340 nm absorbance. The glutamate concentration was calculated as the slope of the absorbance over time compared to a standard curve.

### Statistical analysis

All the data analysis and plot generations were carried out using GraphPad Prism version 7 (GraphPad Software, Inc. USA) and expressed as mean±SEM. Two-way repeated measure ANOVA was applied for MWM experiment, whereas one-way ANOVA was used in other experiments. For the analysis of seizure severity and convulsive behavior, independent t-test was used. Sidak’s correction for multiple comparisons was used as a post hoc test. The minimum significance value was considered as p < 0.05. All experiments were analyzed in a blind fashion.

## Results

### Seizure severity, convulsive behavior and animal mortality

**Fig 2:**
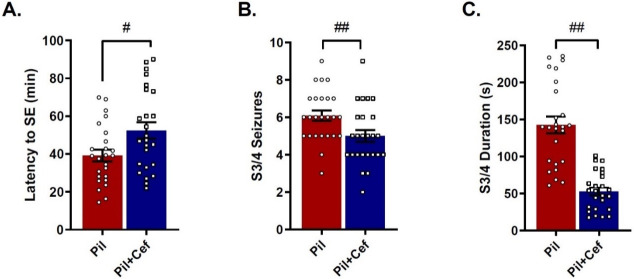
Positive effect of ceftriaxone treatment on seizure severity and convulsive behavior in animal models of temporal lobe epilepsy. The latency to the start of SE (A), number of stage 3 and 4 seizures (B) and average duration of stage 3 and 4 seizures (C) were shown. All data are shown as mean±SEM. # p<0.05, ## p<0.01 compared to the Pil group.

Among animals which received pilocarpine (Pil), 23% (n = 17) died during the course of epileptogenesis and 7% (n = 5) of the animals did not develop SE (Status epilepticus), which were not included in the experiments. Independent t-test analysis showed a significant difference in SE latency (p = 0.0154, n = 25, Fig 2A), as well as number (p = 0.0133, n = 25, Fig 2B) and duration (p < 0.0001, n = 25, Fig 2C) of S3 and S4 seizures between Pil and Pil+Cef groups, demonstrating that ceftriaxone administration could reduce seizure severity (Fig 2). Although SE development did not differ among the groups, the mortality rate was significantly higher in the animals of the Pil group (p = 0.0451, n = 40–51).

### Gene expression

**Fig 3:**
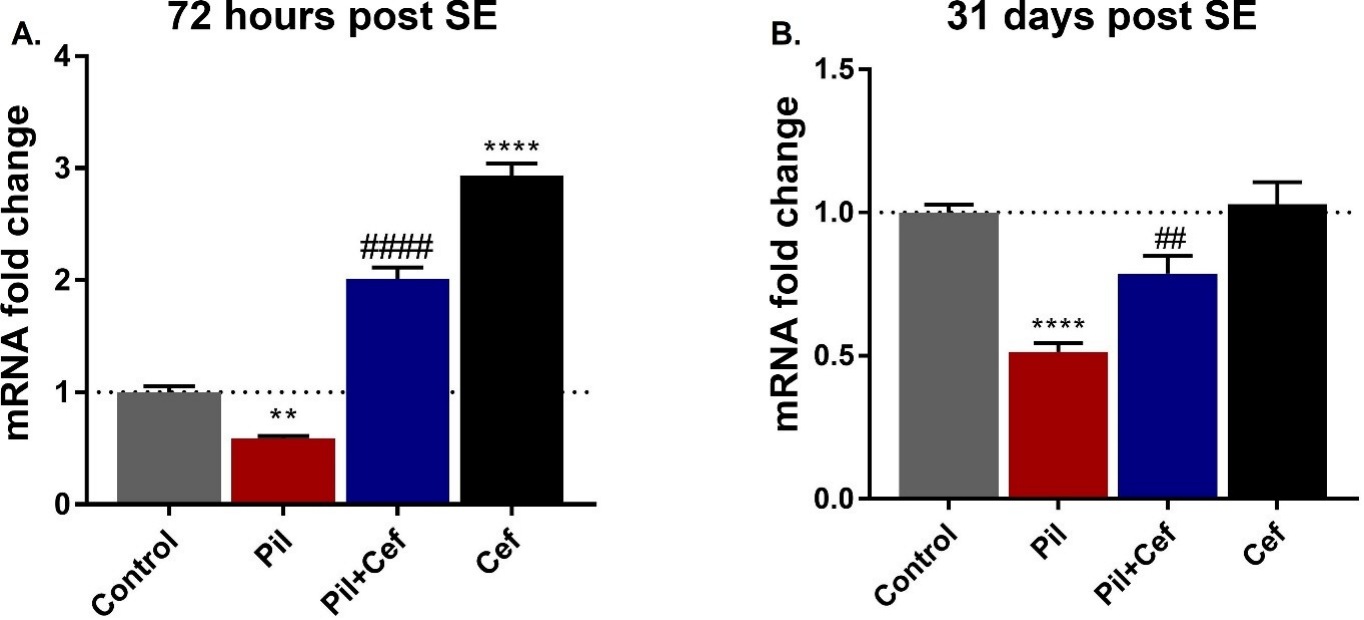
GLT-1 Expression in the animal groups studied in the acute (72 hours post SE) and chronic (31 days post SE) phase of epileptogenesis in the hippocampus. Ceftriaxone treatment could increase the level of GLT-1 expression as compared to the untreated epileptic animals (Pil group) both 72 hours (A) and 31 days (B) after model induction. All data are shown as mean±SEM. ** p<0.01, **** p<0.0001 as compared to the Control and ## p<0.01, #### p<0.0001 as compared to the Pil group.

The hippocampal mRNA level of GLT-1 72 hours (F_3, 16_ = 178.9, p < 0.0001, Fig 3A) and 31 days (F_3, 16_ = 20.15, p < 0.0001, Fig 3B) post SE was analyzed using one-way ANOVA statistical test (Fig 3). Sidak’s multiple comparison revealed a significant decline in the GLT-1 mRNA expression level in the Pil group, both 72 hours (p = 0.0058, dF = 16, n = 5, Fig 3A) and 31 days (p < 0.0001, dF = 16, n = 5, Fig 3B) post SE. Ceftriaxone treatment of pilocarpine administered animals in the Pil+Cef group exhibited a significant increase in the GLT-1 mRNA expression level compared to the Pil group 72 hours (p < 0.0001, dF = 16, n = 5, Fig 3A) and 31 days (p = 0.0066, dF = 16, n = 5, Fig 3B) post SE. Ceftriaxone treatment alone in the Cef group significantly elevated the GLT-1 mRNA 72 hours post SE (p < 0.0001, dF = 16, n = 5, Fig 3A), but did not show any long-term effect on the level of GLT-1 expression 31 days post SE (Fig 3B).

### Glutamate content

**Fig 4:**
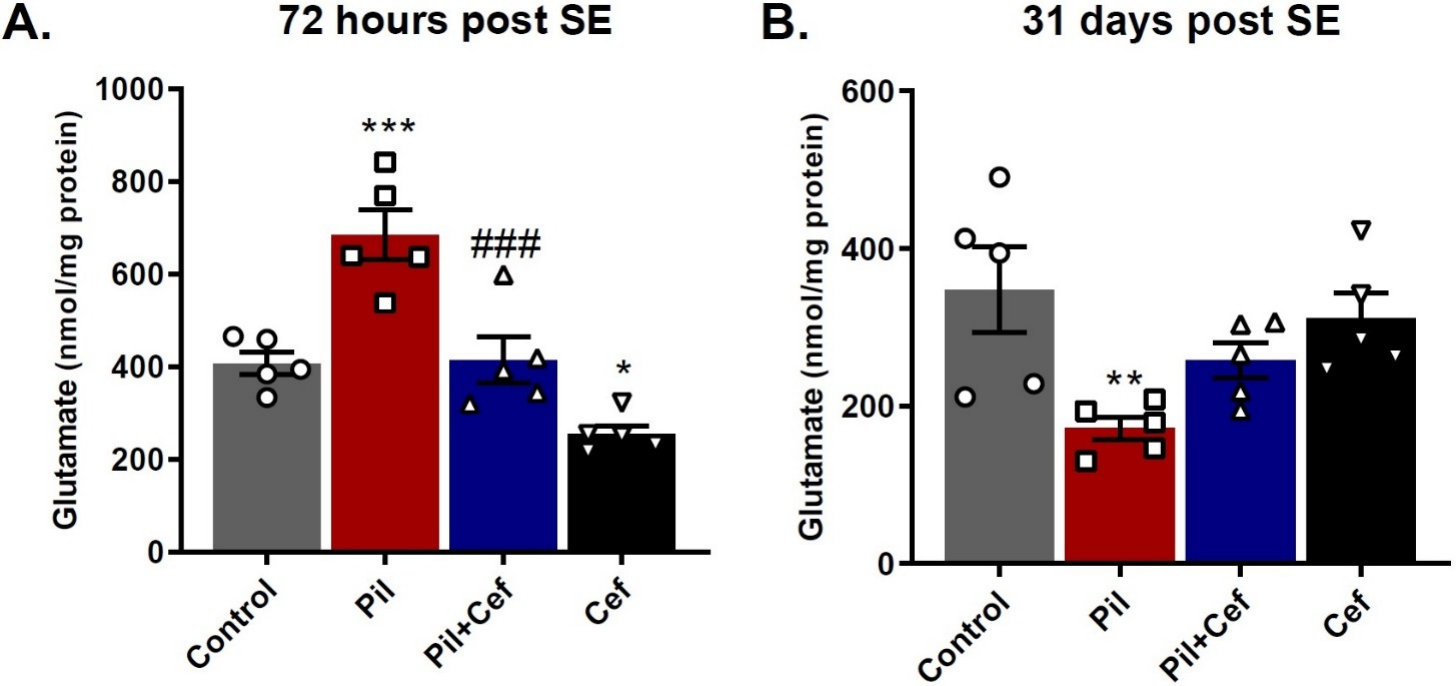
The effect of temporal lobe epilepsy and ceftriaxone treatment on the total glutamate concentration in the hippocampus in the acute (A) and chronic phase of epileptogenesis (B). Ceftriaxone treatment of epileptic animals (Pil+Cef group) could decrease glutamate content 72 hours post SE as compared to the untreated epileptic animals. All data are shown as mean±SEM. * p<0.05, ** p<0.01, *** p<0.001 as compared to the Control and ### p<0.001 as compared to the Pil group.

The total glutamate content of the hippocampus 72 hours and 31 days post SE was evaluated (Fig 4). One-way ANOVA showed a significant difference among the groups both 72 hours (F_3, 16_ = 20.45, p < 0.0001, Fig 4A) and 31 days (F_3, 16_ = 4.945, p = 0.0129, Fig 4B) post SE. Sidak’s multiple comparison demonstrated that 72 hours post SE, glutamate content in the Pil group was significantly higher than the control group (p = 0.0004, dF = 16, n = 5). Animals in the Pil+Cef group also showed a significant decrease in the glutamate content as compared to their control counterparts (p = 0.0005, dF = 16, n = 5). Animals who received Ceftriaxone alone, in the Cef group, showed a significant decline in the glutamate content in comparison to the Control group (p = 0.0441, dF = 16, n = 5) (Fig 4A). In the chronic phase of epileptogenesis, 31 days post SE, Sidak’s multiple comparison revealed a significant decline in the glutamate content of the animals in the Pil group compared to the control animals (p = 0.0062, dF = 16, n = 5, Fig 4B).

### Glutamine synthetase (GS) activity

**Fig 5:**
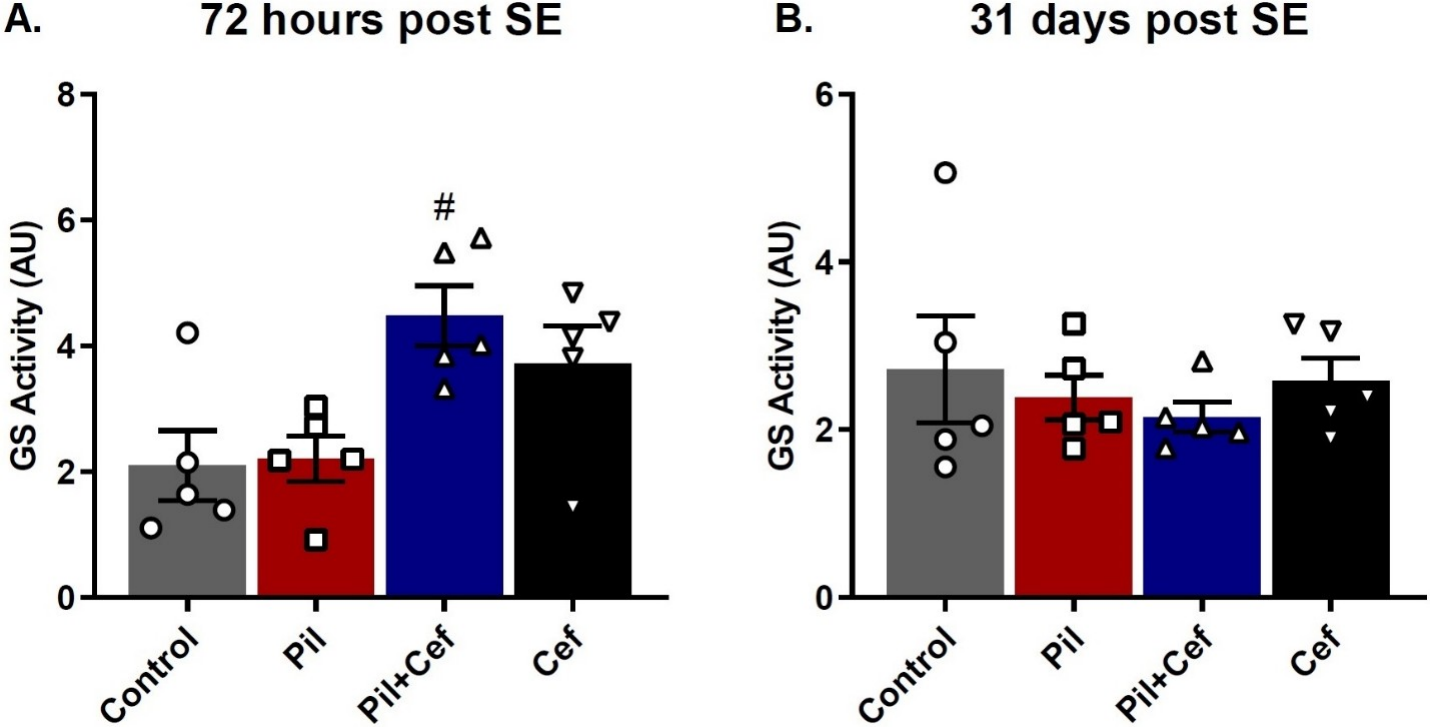
Glutamine Synthetase (GS) activity in the hippocampus of animals studied 72 hours and 31 days post SE. Ceftriaxone treatment increase the enzyme activity in the animals of Pil+Cef group as compared to the Pil group during the acute phase of epileptogenesis. All data are shown as mean±SEM. # p<0.05 as compared to the Pil group.

GS activity in the hippocampus was evaluated 72 h and 31 days post SE (Fig 5). One-way ANOVA test revealed no significant difference in the GS activity 31 days post SE among the groups (F_3, 16_ = 0.4183, p = 0.7423). Data on GS activity 72 hours post SE were analyzed using one-way ANOVA test which showed a significant difference between the groups (F_3, 16_ = 5.331, p = 0.0097). Sidak’s multiple comparison demonstrated that the activity of this enzyme was significantly higher in the Pil+Cef group compared to the Pil group (p = 0.0174, dF = 16, n = 5).

### Morris water maze

**Fig 6:**
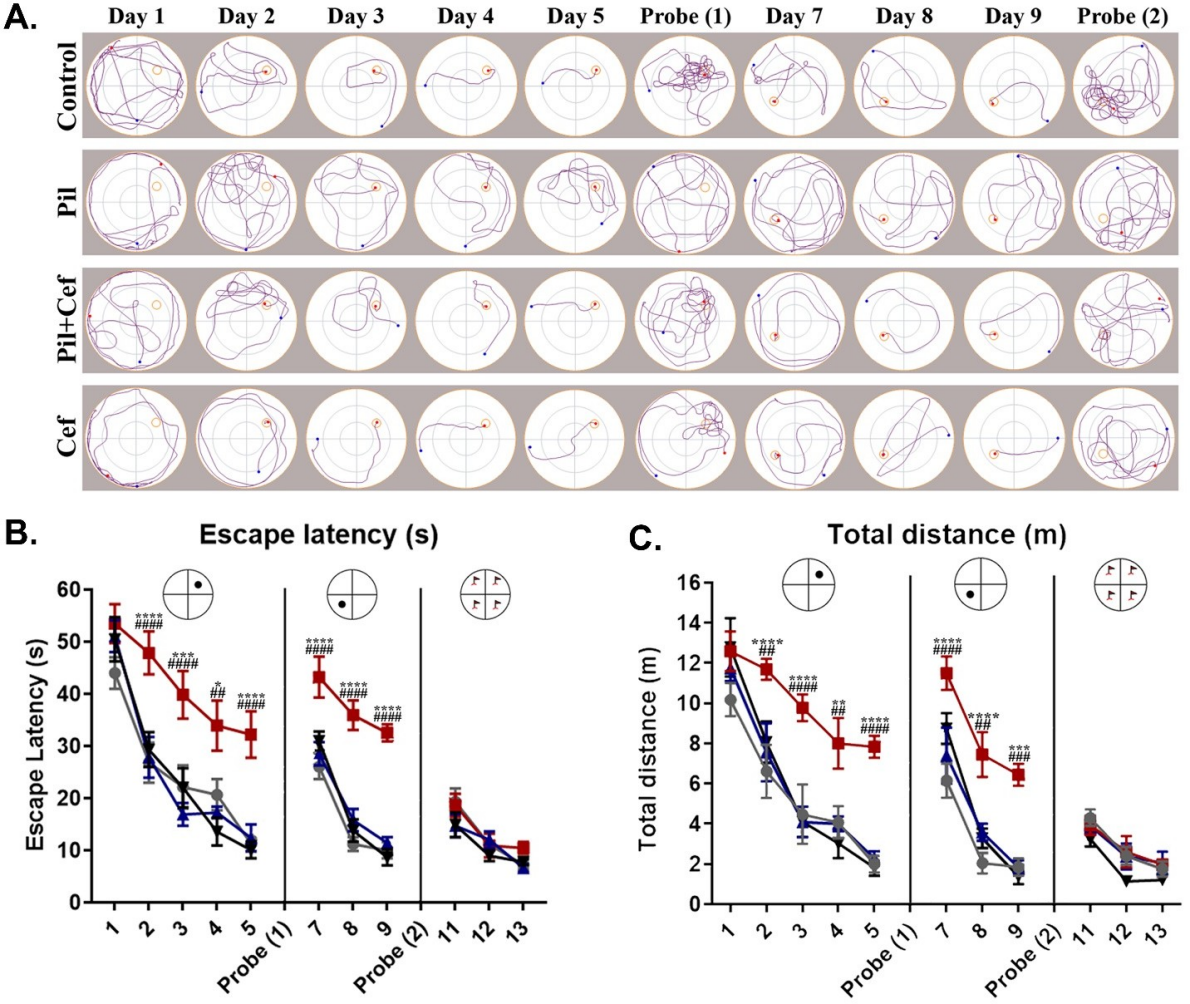
Spatial learning in the morris water maze navigation task in animals studied 31 days post SE. The track plots during the course of learning, reversal learning and visible platform trials illustrate the impaired cognitive functions in the animals of the Pil group, while animals in the Pil+Cef group showed a better performance (A). Data on the time to find the escape platform (escape latency) demonstrated the same results (B). Also, this was obvious in the total distance travelled during the trials (C). All data are shown as mean±SEM. * p<0.05, ** p<0.01, *** p<0.001, **** p<0.0001 as compared to the Control and ## p<0.01, ### p<0.001, #### p<0.0001 as compared to the Pil group.

MWM test was used to investigate spatial learning and memory (Fig 6A). Escape latency (Fig 6B) on different days were analyzed by two-way ANOVA with repeated measures and significant differences (F_3, 24_ = 32.11, p < 0.0001) was observed (Fig 6B). Tukey’s post-hoc test showed that the escape latency in the Pil group was significantly higher compared to the control group during initial and reversal learning phases of the MWM test (p < 0.05, n = 7). However, ceftriaxone treatment in Pil+Cef group was able to significantly decrease the escape latency and subsequently rescue the cognitive impairments following Pil administration (p < 0.01, n = 7). The same results were obtained for the swim distance during the trials (Fig 6C). The mean swimming speed of the animals was recorded to investigate locomotor activity. The two-way ANOVA test with repeated measures did not show any difference between the study groups (Data are not shown).

**Fig 7:**
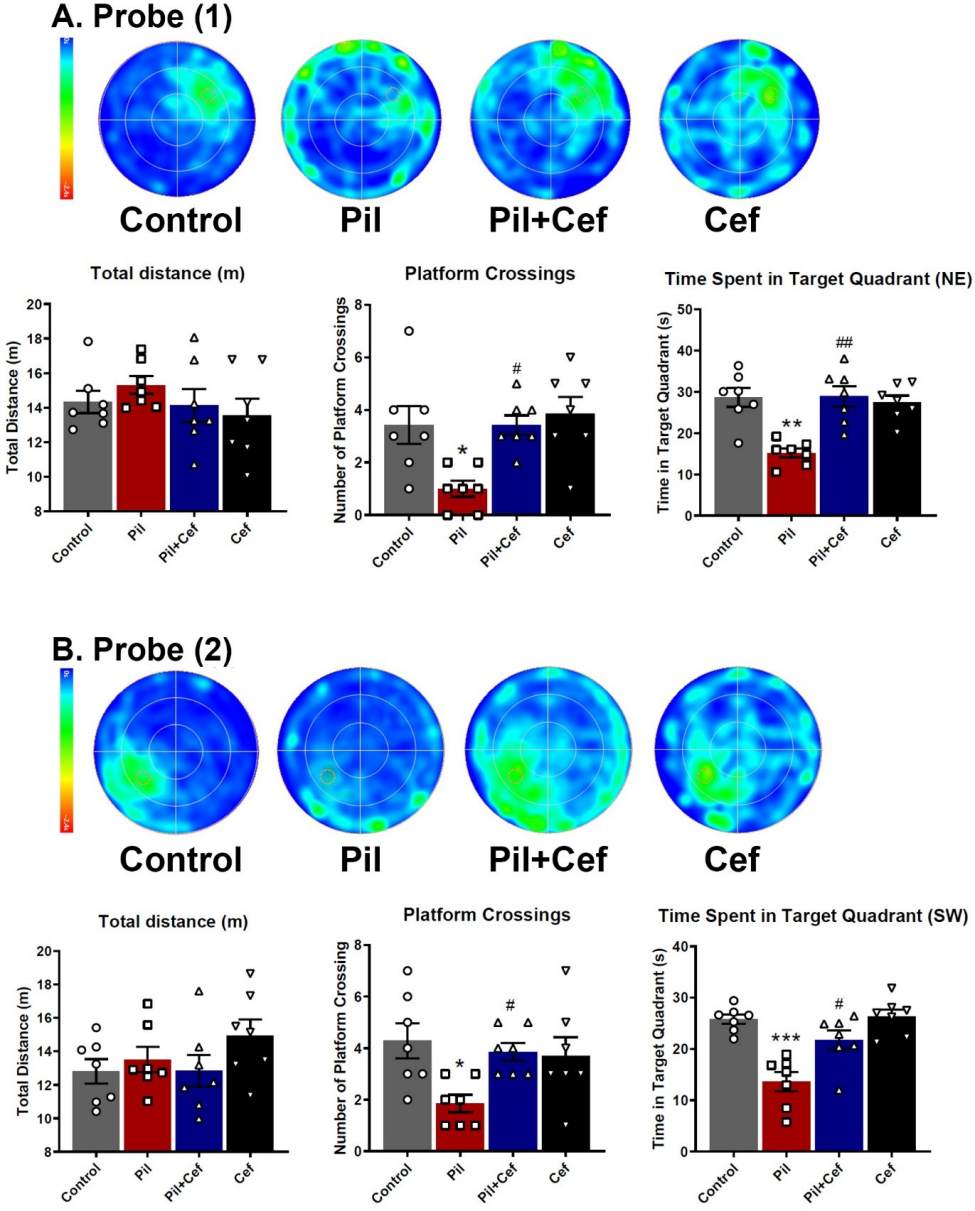
The mean heatmaps and studied parameters during the first (A) and second (B) probe trials. Temporal lobe epilepsy induction impaired the memory functions of the animals in both probe trials, while the Ceftriaxone treatment could alleviate the memory loss. All data are shown as mean±SEM. * p<0.05, ** p<0.01, *** p<0.001, **** p<0.0001 as compared to the Control and # p<0.05, ## p<0.01, ### p<0.001as compared to the Pil group.

To investigate the reference memory, a probe test was carried out on the sixth day (Fig 7). The data acquired was analyzed using one-way ANOVA followed by Sidak’s multiple comparison. One-way ANOVA revealed a significant difference among the groups in the following parameters: the number of platform crossings (F_3, 24_ = 5.893, p = 0.0037), and the time spent in the target quadrant—the quadrant where the platform was located during the acquisition phase (F_3, 24_ = 11.52, p < 0.0001). Sidak’s multiple comparison showed that animals in the Pil group had a significantly lower number of platform crossings (p = 0.0114, dF = 24, n = 7), and the time spent in the target quadrant (p = 0.0002, dF = 24, n = 7) as compared to the animals of the Control group. Ceftriaxone treatment of animals in the Pil+Cef group resulted in a significant increase in number of platform crossings (p = 0.0114, dF = 24, n = 7) and the time spent in the target quadrant (p = 0.0001, dF = 24, n = 7) compared to the animals of the Pil group (Fig 7A).

Another probe trial was taken in day 10 to assess the memory of the animals after reversal learning (Fig 7B). One-way ANOVA revealed a significant difference among the groups in the following parameters: number of platform crossings (F_3, 24_ = 3.842, p = 0.0223) and the time spent in the target quadrant (F_3, 24_ = 14.48, p < 0.0001). Sidak’s multiple comparison test showed a significant decreased in number of platform crossings (p = 0.0136, dF = 24, n = 7) and the time spent in the target quadrant (p < 0.0001, dF = 24, n = 7) among the animals in the Pil group compared to the Control group. Animals in the Pil+Cef group demonstrated a significant elevation in both the number of platform crossings (p = 0.0487, dF = 24, n = 7) and the time spent in the target quadrant (p = 0.0032, dF = 24, n = 7) compared to the animals in the Pil group.

### Novel object recognition task (NORT)

**Fig 8:**
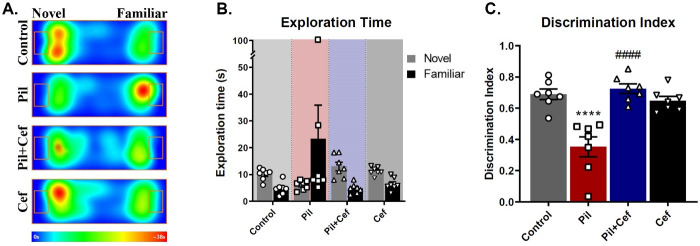
Positive effect of Ceftriaxone treatment on the object recognition memory of epileptic animals. The heatmaps illustrate the average location of the animals during the testing trial (A). The exploration time of novel and familiar objects were not different among the groups (B), while Ceftriaxone treatment of epileptic animals increased the discrimination index as compared to their epileptic untreated counterparts (C). All data are shown as mean±SEM. **** p<0.0001 as compared to the Control and #### p<0.0001 as compared to the Pil group.

Novel object recognition task (NORT) was used to check object recognition memory. The recognition of novelty requires more cognitive skills. Therefore, novel object exploration time, discrimination index and total time of object exploration were assessed (Fig 8).

One-way ANOVA of the novel object exploration time showed a significant difference among the groups (F_3, 24_ = 8.812, p = 0.0004). The Sidak’s multiple comparison revealed that the animals in the Pil group spent significantly less time exploring the novel object as compared to the control group (p = 0.0234, dF = 24, n = 7), while Ceftriaxone treatment in the Pil+Cef group could elevate this parameter compared to the Control group (p = 0.0001, dF = 24, n = 7). Discrimination index (ratio of the novel object exploration time to the total exploration time) were analyzed using one-way ANOVA and a significant difference among the groups was observed (F_3, 24_ = 16.77, p < 0.0001). Sidak’s multiple comparison showed that this index was significantly decreased in Pil group compared to the control animals (p < 0.0001, dF = 24, n = 7). The animals in the Pil+Cef group, however, demonstrated a significant increase in this parameter as compared to the Control group (p < 0.0001, dF = 24, n = 7). Findings related to the total exploration time were analyzed using one-way ANOVA and no significant deference among the groups was detected.

## Discussion

Many studies have shown that temporal lobe epilepsy causes learning and memory impairments. One of the major contributing factors to these impairments is the duration of SE (Status epilepticus) after pilocarpine injection. In this study, SE was inhibited by diazepam 90 minutes after initiation. Chen et al. In 2013 showed that SE can lead to serious cognitive impairments associated with spontaneous seizures as well as mossy fiber sprouting (MFS) [28] which results in increased excitatory load in the dentate gyrus (DG) and subsequently CA3 [29]. In addition to neurons and neuronal pathways, activation of astrocytes and astrogliosis is a prominent feature in brain tissue in models of epilepsy [30,31] and is one of the possible mechanisms of cognitive impairments in the TLE models [32]. Many studies have shown that targeting astrocytes can reduce epilepsy-related cognitive impairments [32–34].

We used chronic injection of ceftriaxone to increase the expression of GLT-1 in astrocytes. Many studies have shown that injecting ceftriaxone at a dose of 200 mg/kg/day chronically (for 5 days) can increase GLT-1 protein and gene expression [14,35–37]. Ceftriaxone was injected in a manner that, according to a previous study [38], the highest expression of GLT-1 occurred in the acute period of pilocarpine (initial 72 h post-SE, with highest level of mRNA after 3^rd^ injection). MWM test results showed that treatment with ceftriaxone (Pil+Cef group) can improve pilocarpine-induced cognitive impairment, compared to the epileptic animals (Pil group). In addition to direct learning, reversal learning was also improved in this group. The findings of this study are in line with the results of Hota et al., who reported the positive effects of ceftriaxone on hypoxia-induced excitotoxicity, memory and learning [39]. Increasing GLT-1 expression and decreasing excitotoxicity can prevent over-stimulation of hippocampal neural circuits and possibly reduce excitability of their neurons. Vinet et. al (2018) have noticed the presence of an ischemic-like lesion in the CA3 lacunosum-moleculare following pilocarpine-induced SE. Ceftriaxone treatment may exert some of its positive effects through inhibition of such lesion formations in the hippocampus [40].

In addition to spatial memory, object recognition memory was studied using the Novel Object Recognition Task (NORT) that depends on the health of the medial temporal lobe (mTL) [41]. The results of this study showed that epileptic animals receiving pilocarpine tend to spend less time exploring the novel object. This impairment was rescued upon Ceftriaxone treatment. Given that the total time of exploration did not differ between the study groups, it can be concluded that the exploration activity as well as the motivation to examine objects in all animals were the same. Object recognition memory is dependent on the hippocampus and is impaired by the destruction of the hippocampal neurons [42–44]. Numerous studies have identified object recognition memory degradation after epilepsy induction [45,46]. As noted, treatment with ceftriaxone can prevent the excitotoxicity and reactivation of astrocytes by increasing GLT-1 expression and affect the excitability of the neural circuitry.

We measured the expression of GLT-1 transporter mRNA in hippocampal tissue by RT-qPCR 72 hours and 31 days post SE. As expected, ceftriaxone treatment alone (Cef group) increased GLT-1 expression 72 h post SE. It was found that expression of this transporter in epileptic animals decreased 72 h post SE and treatment with ceftriaxone (Pil+Cef group) was able to increase mRNA levels of this gene compared to the Pil group. These findings indicated that in addition to increased neuronal excitability, decreased glutamate uptake by GLT-1 transporter in epileptic animals could aggravate the excitotoxic conditions. Studies have shown that GLT-1 expression after SE changes over time, which is visible at both the mRNA and protein levels [47]. Our observed were consistent with the findings of Hubbard et al., who showed that GLT-1 expression was significantly reduced 4 days post SE [47]. According to the results of the present study, GLT-1 expression also remained low 31 days post SE, whereas its expression level in the Cef group who received ceftriaxone alone was normal and equivalent to the control. On the other hand, epileptic animals treated with ceftriaxone (Pil+Cef group) had an increased GLT-1 expression 72 h and 31 days post SE, indicating a long-term effect on astrocytes and hippocampal tissue of epileptic animals. The mechanism of the effect of ceftriaxone on GLT-1 gene expression is not well understood. However, it seems that ceftriaxone enhances transcription and mRNA expression of this gene by affecting NF-κB transcription factor [15].

As stated, in epileptic conditions where GLT-1 levels decrease, it is not unexpected that glutamate-glutamine cycle is impaired and the glutamine required for the proper functioning of neurons is not produced.

In the present study, total glutamate content in the hippocampal tissue were assessed in the acute phase of epilepsy (72 hours post SE) as well as in the chronic phase of epilepsy (31 days post SE induction). The findings showed that in the acute phase, glutamate levels elevated in epileptic animals (Pil group) compared to the Control. Animals who received ceftriaxone in addition to pilocarpine (Pil+Cef group) had lower levels of glutamate in the hippocampal tissue than their epileptic counterparts. However, in the chronic phase of epilepsy, glutamate levels in epileptic animals were significantly reduced compared to control animals and ceftriaxone treatment had no effect on this decrease.

In line with this finding, previous studies have shown that in the acute phase of epilepsy, glutamate levels in the hippocampal tissue increase significantly [48]. This increase is likely due to the increased production of glutamate from glucose in the citric acid cycle (TCA) following the severe need for neurons after continuous stimulations. Given the effects of ceftriaxone on GLT-1 expression, it can be deduced that increased astrocytic uptake of glutamate can help improve the glutamine-glutamate cycle and decrease glutamate levels by converting glutamate to glutamine and GABA. As the data for GLT-1 gene expression indicate, the acute phase of epilepsy is accompanied by a decrease in GLT-1 expression, which disrupts glutamate turnover.

In contrast, findings related to the chronic phase of epilepsy (31 days post SE) showed that glutamate levels in the hippocampus of epileptic animals decrease. Previous studies have demonstrated that impaired cellular metabolism can decrease glutamate in epileptic patients [49]. Given the lack of a correlation between glutamate levels and cell death in cellular studies [50], glutamate depletion is likely due solely to impaired cellular metabolism (such as substance transport, reduced TCA cycle activity, and many mitochondrial damages) [51]. It should be noted that with no effect of ceftriaxone treatment on glutamate levels, ceftriaxone probably exerts its positive effects only indirectly and has little effect on improving cell metabolism.

In conclusion, it seems that ceftriaxone treatment can alleviate the cognitive impairments and reduce (at least to some extent) the cellular and molecular deficits caused by epileptic seizures.

## Supporting information

S1 Data (XLSX)
